# “You only live twice”: multidisciplinary management of catastrophic case in placenta Accreta Spectrum-a case report

**DOI:** 10.1186/s12884-020-2817-2

**Published:** 2020-02-28

**Authors:** David Atallah, Hicham Abou Zeid, Malak Moubarak, Maya Moussa, Nadine Nassif, Victor Jebara

**Affiliations:** 1 Saint Joseph University, Beirut, Lebanon; 2 Department of Obstetrics and Gynecology, Hôtel-Dieu de France University Hospital, P.O. Box: 116-5137, Beirut, Lebanon; 3 Department of Anesthesiology, Hôtel-Dieu de France University Hospital, Beirut, Lebanon; 4 Department of Cardiovascular Surgery, Hôtel-Dieu de France University Hospital, Beirut, Lebanon

**Keywords:** Placenta accreta spectrum (PAS), Cardiac arrest, Pulmonary embolism, Intraventricular thrombus, Transesophageal ultrasound

## Abstract

**Background:**

Placenta percreta is associated with high hemorrhagic risk and can be complicated with fatal thromboembolic events. Involving a multidisciplinary team in the treatment of these patients is mandatory to reduce morbidity and mortality.

**Case presentation:**

This paper reports the case of a 22-year-old patient with placenta percreta who was referred to our tertiary care center for delivery. Few hours after undergoing a successful cesarean hysterectomy, the patient developed a pulmonary embolism and cardiac arrest. A transthoracic echocardiogram done in the intensive care unit (ICU) showed a thrombus in the right ventricle. After cardiac resuscitation, the patient underwent an urgent thoracotomy and a pulmonary artery thrombectomy; many clots were retrieved from the pulmonary artery. After weaning from extracorporeal circulation, an intraoperative transesophageal cardiac ultrasound enabled the medical team to detect a new free-floating thrombus in the right atrium and right ventricle, and consequently to perform an embolectomy and prevent the patient’s death.

**Conclusion:**

This case emphasizes the role of multidisciplinary team in treating high-risk obstetric cases that could be complicated with massive and fatal thromboembolic events. The use of intraoperative transthoracic echocardiography helps in detecting a new thrombus and guides the anesthesiologist in the intra-operative monitoring.

## Background

Placenta percreta patients are at high risk for life-threatening hemorrhage. Unfortunately, these cases are also at risk of massive and fatal thromboembolic events. Therefore, reducing morbidity and mortality requires the referral of these cases to a tertiary care center and the involvement of a multidisciplinary team.

## Case presentation

We report the case of a 22-year-old pregnant women, G2P1, diagnosed with placenta accreta spectrum (PAS) and referred to our institution at 31 weeks of gestation for further care and management. Earlier, at 25 weeks of gestation, the patient reported vaginal spotting. An ultrasound performed by her primary obstetrician was suggestive of a placenta percreta. At 30 weeks, she experienced a preterm premature rupture of membranes and moderate vaginal bleeding requiring her admission in a primary care hospital. During her stay, she received tocolytics, antibiotics and steroids. Strict bed rest was prescribed but no thrombosis prophylaxis was administered given her history of vaginal bleeding that lasted one week. Upon confirming the diagnosis of placenta previa with accreta spectrum on a pelvic MRI, the patient was referred to our tertiary care center to schedule her delivery.

Eight months earlier, she underwent a cesarean section due to a protracted labor. Postoperatively, she received no prophylactic anticoagulation. Besides, she was taking oral contraceptives for three months because of a persistent vaginal bleeding; she stopped them three months before pregnancy. The patient reported no relevant past medical or surgical events. Her father died at the age of 42 of an ischemic stroke and two uncles had histories of non-specific thromboembolic events.

On admission, the patient was afebrile, hemodynamically stable and did not complain of pelvic pain. She noted only light to moderate persistent vaginal bleeding. Urgent ultrasound showed a viable fetus with appropriate biometrical parameters and no amniotic fluid. Fetal cardiotocography revealed regular uterine contractions. She underwent urgent delivery by cesarean section and hysterectomy under general anesthesia according to our specially developed technique [1, 2]. The placenta was previa, anteriorly located and slightly lateralized to the left and reached the uterine serosa without perforating it. The placenta was bulging under a thin uterine serosa with a lot of neo-vascularization at this level.

The total estimated blood loss during surgery was 1800 ml. This large amount of intraoperative bleeding was essentially from the vagina, which was unfortunately uncontrollable before removing the uterus completely. The anesthesiologist in charge had to transfuse the patient with allogenic red blood cells (RBC; 7 units), fresh-frozen plasma (FFP; 6 units) and platelets (1 unit) to maintain her hemodynamic stability. Since more than four RBC units were transfused in less than one hour, the transfusion was considered massive and 1:1 ratio of FFP to RBC scheme was used. At the end of surgery and transfusion, an evaluation of complete blood count (CBC) revealed a hematocrit level of 32% and hemoglobin level of 10.8 g/dl. The patient was also normothermic and hemodynamically stable. She was waked up from anesthesia and was transferred to ICU for postoperative surveillance. At ICU admission, she was hemodynamically stable with normal neurologic examination.

**Fig. 1:**
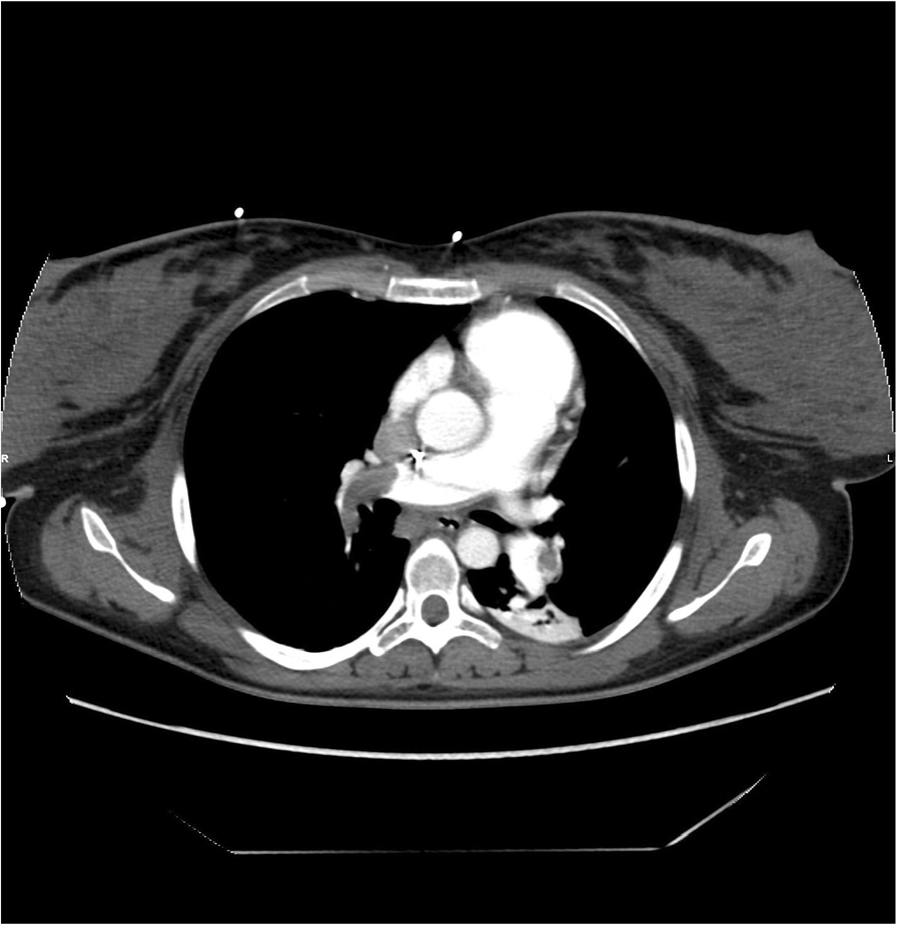
Pulmonary CT angiogram. The CT angiogram shows many intraluminal filling defects suggestive of massive bilateral pulmonary embolism

Two hours later, the patient became cyanotic and went into cardio pulmonary arrest. Cardiac monitoring showed pulseless ventricular tachycardia. Arterial blood gases showed hypocapnia (PaCO_2_ of 30 mmHg) and hypoxia (PaO_2_ of 61 mmHg). Characteristic S1Q3 wave detected on electrocardiography was associated with hypoxia and hypocania, which were highly suggestive of pulmonary embolism (PE). She was intubated and received 40 min of cardiopulmonary resuscitation. A transthoracic echocardiogram done in ICU showed a thrombus in the right ventricle. Urgent pulmonary angiogram after hemodynamic stabilization confirmed the diagnosis of bilateral massive PE (Fig. 1).

**Fig. 2:**
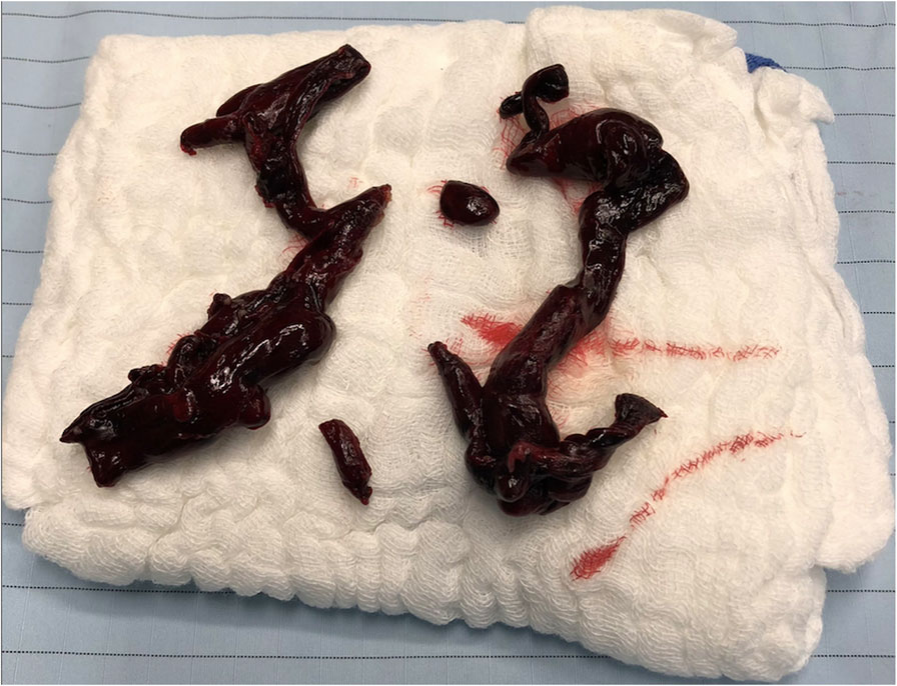
removed clots. A pulmonary artery thrombectomy was performed and many clots were retrieved from the pulmonary artery

So the patient underwent an urgent thoracotomy and an extracorporeal circulation was used. During surgery, a simultaneous transesophageal cardiac ultrasound was done showing a dilated right ventricle containing clots. A pulmonary artery thrombectomy was performed and multiple clots were retrieved from the pulmonary artery (Fig. 2).

Once the situation was stabilized, an inferior vena cava filter was placed via an abdominal incision and external iliac vein catheterization. After weaning from extracorporeal circulation, an intraoperative transesophageal cardiac ultrasound revealed the presence of a new free-floating thrombus in the right atrium and right ventricle (Video). A second extracorporeal circulation using new cannulation of the large vessels was initiated and a second embolectomy was performed. The patient was transferred to the cardiac surgery care unit (CSU) for surveillance. While at CSU, she was placed on therapeutic intravenous heparin for anticoagulation and had compressive pneumatic stockings in place at all times.

Two days later, the patient experienced a major hemoperitoneum with distended abdomen and tachycardia due to massive anticoagulation that necessitated a second laparotomy to achieve hemostasis. Only blood clots were found in the peritoneal cavity without active source of bleeding. All vascular pedicles were controlled and the vaginal cuff was re-sutured. Intraoperative evaluation of CBC revealed a hematocrit level of 26% and hemoglobin level of 8.7 g/dl. Intraoperative allogeneic RBC (4 units) and FFP (5 units) were transfused. The rest of her post-operative course was uneventful.

The patient was discharged on postoperative day 13. She received acenocoumarol per os for a lifetime anticoagulation. A follow-up was organized with a hematologist to perform a full thromboembolic disease work-up. A panel of tests for hypercoagulability was performed and showed: protein S deficiency, presence of circulating lupus anticoagulant, heterozygous methylenetetrahydrofolate reductase (MTHFR) mutation and genotypes of plasminogen activator inhibitor (PAI), human platelet antigen (HPA), angiotensin converting enzyme (ACE), and apolipiprotein E (Apo E) with a moderate risk for thrombosis.

The pathologic report confirmed the diagnosis of abnormally invasive placenta type percreta (grade 3a, according to FIGO classification): hysterectomy specimen showing villous tissue reaching the uterine serosa and in multiple places breaching the serosa and invading the extrauterine adipose tissue with a fibrous reaction at this level.

## Discussion and conclusion

Maternal cardiac arrest is a very complex and demanding situation that requires the intervention of a multidisciplinary well-trained team [3]. Unfortunately, the incidence of maternal cardiac arrest is rising according to recent reports from the Netherlands, the United States and the United Kingdom [4–6]. The most common causes of maternal cardiac arrest are PE (24%), major obstetric hemorrhage (18%) and amniotic fluid embolism (16%) [3]. Other causes are severe preeclampsia and eclampsia, septic shock, complications of anesthesia and cardiac diseases [5–7]. Also, cardiac arrests occurring in emergency or in operating rooms are associated with higher maternal survival rates than those occurring in delivery rooms and maternity wards [7].

Pregnancy and postpartum are the most high-risk periods for venous thromboembolism events (VTE), mainly deep vein thrombosis, PE and cerebral thromboembolism. According to a French study on maternal deaths, PE is responsible of 9% of maternal deaths with 54% of these deaths occurring in the postpartum period [8]. Essentially, the risk of thromboembolic events seems to be more elevated in the postpartum period since more inflammatory and traumatic risk factors including cesarean section, postpartum hemorrhage and resuscitative hysterectomy, are associated with a pregnancy favorable background. According to a recent Cochrane review, no sufficient evidence is available to guide recommendations for thromboprophylaxis during pregnancy and during the postnatal period [9]. Despite an existing validated risk-stratification system for VTE in pregnancy and postpartum, it remains unclear whether a pharmacological and/or mechanical prophylaxis should be given for a high-risk parturient [10].

In this report, we tried to point out an unusual and insidious finding that occurred shortly after the delivery of a patient with placenta percreta. During cardiopulmonary resuscitation and given the sudden onset of events, a PE was highly suspected. After stabilization, an urgent pulmonary angiogram showed a bilateral massive PE. Indeed, diagnostic imaging should not be withheld nor postponed in pregnant or non-pregnant patients with suspected PE because of the fatal consequences of a misdiagnosis [11].

In our case, the patient presented multiple risk factors that could have contributed to the development of PE. The list includes immobilization and bed rest in the previous hospitalization, lack of prophylaxis for an immobilized pregnant patient with placenta percreta, family history, prolonged surgery, hypercoagulable state of pregnancy and previous use of contraceptive. Placenta percreta is an important factor not to be underestimated since a bulging placenta occupying the pelvis will promote a vein stasis and consequently the formation of vein thrombosis. Also, a general consensus is reached on the effectiveness of mechanical prophylaxis at reducing rates of VTE in obstetric patients with at least one large study showing a reduction in fatal PE [10]. However, it was not sufficient to prevent these events in the present case. Besides, due to the vaginal spotting in the PAS setting, a pharmacological anticoagulation was unfortunately contraindicated which might have contributed to the patient’s PE.

This case presentation is one of few cases of thromboembolic events in patients with placenta accreta. These cases are usually underreported because they are associated with a higher mortality rate. The first one to be mentioned was reported in late 1960s where a syncytial trophoblastic PE was presented in a patient with placenta increta and preeclampsia [12]. Like other types of emboli, a trophoblastic embolism can lead to catastrophic consequences causing sudden death [12–14]. Therefore, awareness of this syndrome and prompt action are necessary to prevent tragic consequences [13]. Also, the presence of placenta percreta may increase the risk of amniotic fluid embolus, as suggested by Styron et al. in their case presentation [15].

While some manifestations of PE will be limited to hypoxia, hypocapnia, and tachycardia, others will present suddenly with a cardiac arrest as in the present case. In contrast, other colleagues reported a case of cardiac arrest caused by PE preoperatively in a patient with placenta previa accreta who underwent a cesarean section immediately after cardiopulmonary resuscitation [16]. Using abdominal ultrasound, they demonstrated the presence of floating thrombus in the inferior vena cava [16]. A recently reported case showed similar findings of an incidentally found inferior vena cava thrombus by using an intraoperative transesophageal echocardiography (TEE) [17]. Similarly, an inferior vena cava filter was placed via an abdominal incision and external iliac vein catheterization since percutaneous vascular access was impossible and the operating room table was radio-opaque. Inferior vena cava filter placement aimed to prevent further embolic events.

TEE is a monitoring tool that helps to reveal the presence of new thrombus, enabling the operative team to be particularly vigilant for a PE [17]. To our knowledge, our case is the first reported case in the literature of an incidentally found thrombus in the right ventricle using TEE. TEE was performed simultaneously during the thoracotomy and had enabled us to detect at the end of the procedure the presence of a new free-floating thrombus in the right atrium and right ventricle, requiring the initiation of a second extracorporeal circulation and embolectomy. In fact, the second thrombus could not be detected and could have led to the patient’s death, if no TEE was performed. This is to emphasize the importance of a multidisciplinary team that enabled the early detection of thrombosis, and consequently, an urgent transfer of the patient in the operation room to receive a salvage embolectomy. That’s why these kinds of cases need to be addressed in centers of excellence where expertise and multidisciplinary teams are available to manage the most serious complications [18, 19].

Placenta percreta is not only the surgeon’s nightmare but also involves the anesthesiologist. Aside from hemorrhagic risk, these cases can be complicated with massive and fatal thromboembolic events. A preoperative screening for deep venous thrombosis could be recommended. However, given the lack of convincing evidence and recommendations to prevent thromboembolic events, it is highly required to determine focus and allocate efforts for quality improvements in obstetric health care: patients with placenta percreta should always be referred to tertiary care centers that grant access to multidisciplinary team management. Only then, you can hope “they will live twice”.

## Supplementary information

**Additional file 1: Video S1.** Transesophageal cardiac ultrasound finding. Intraoperative transesophageal cardiac ultrasound revealed the presence of a new free-floating thrombus in the right atrium and right ventricle.

## Data Availability

Data sharing is not applicable to this article as no datasets were generated or analyzed during the current study.

## Abbreviations

CBC: Complete blood count
CSU: Cardiac surgery care unit
FFP: Fresh frozen plasma
ICU: Intensive care unit
PaCO_2_: Partial pressure of arterial carbon dioxide
PaO_2_: Partial pressure of arterial oxygen
PAS: Placenta accreta spectrum
PE: Pulmonary embolism
RBC: Red blood cell
TEE: Transesophageal echocardiography
VTE: Venous thromboembolism events
